# Atomic Layer-Deposited HfAlOx-Based RRAM with Low Operating Voltage for Computing In-Memory Applications

**DOI:** 10.1186/s11671-019-2875-4

**Published:** 2019-02-07

**Authors:** Zhen-Yu He, Tian-Yu Wang, Lin Chen, Hao Zhu, Qing-Qing Sun, Shi-Jin Ding, David Wei Zhang

**Affiliations:** State Key Laboratory of ASIC and System, School of Microelectronics, Fudan University, Shanghai, 200433 China

**Keywords:** Computing in-memory, RRAM, Switching, Implemented

## Abstract

With Moore’s law closing to its physical limit, traditional von Neumann architecture is facing a challenge. It is expected that the computing in-memory architecture-based resistive random access memory (RRAM) could be a potential candidate to overcome the von Neumann bottleneck problem of traditional computers [Backus, J, Can programming be liberated from the von Neumann style?, 1977]. In this work, HfAlOx-based RRAM which is compatible with CMOS technology was fabricated by an atomic layer deposition (ALD) process. Metal Ag and TaN are selected as top electrodes (TE). Experiments show that the Ag/HfAlOx/Pt device has demonstrated advantages as a memory-computing device because of the low set voltage (0.33~0.6 V) which means low power consumption and good uniformity. Based on a Ag/HfAlOx/Pt structure, IMP logic was implemented at high speed by applying a 100-ns high-frequency low-voltage pulse (0.3 V and 0.6 V). After two steps of IMP implementation, NAND can also be obtained.

## Background

For the boundaries between storage and computing, researchers have proposed a series of research programs: high-bandwidth memory, near-memory computing, and neural compression networks. These methods can reduce the time to access the memory, but they could not solve this problem fundamentally. In order to solve this problem fundamentally, the concept of computing in-memory has gained attention worldwide. It is worth noting that a resistive random access memory (RRAM) device has attracted widespread attention as a competitive candidate for the non-von Neumann computing device because of its capability of in-memory computing [1–6]. Computing in-memory devices act as both computing and storage units in the same circuit [7]. It was first proposed in 1971 by Chua [8]. Almost 40 years later, RRAM-based logic operation was first proposed in 2010 [9]. Since then, RRAM-based computing in-memory device has been extensively studied and many methods of implementation have been proposed [10–14]. But as a computing in-memory device, the most crucial feature is stability and low energy consumption. There are still many issues in this area that need to be explored. In this letter, two kinds of RRAM devices were constructed and the electrical properties were tested. In the process of implementing logic operations, stable set and reset voltages and good uniformity between devices are very important indicators.

So far, a wide variety of materials have shown RRAM behaviors, but few of them were compatible with CMOS process. The binary high-k oxides HfAlOx film was deposited using atomic layer deposition (ALD). ALD is well-suited for deposition of oxide films and over layers for various devices and applications [15] because it is based on surface saturation and precise precursor dosage is not necessary. HfAlOx could be well compatible with the traditional CMOS process and used as the dielectric layer of in-memory computing device. The Ag/HfAlOx/Pt RRAM devices were used to implement stateful logic operations. The IMP logic was regarded as one of four fundamental logic operations (OR, AND, NOT, and IMP) by Whitehead and Russell in 1910 [16]. Moreover, the NAND logic can be obtained by two steps of IMP logic. The NAND logic is known as the universal logic, which means any Boolean logics can be constructed through the NAND logic. This CMOS-compatible, high-speed, and low operation voltage in-memory computing device shows an effective way to solve the traditional von Neumann structure difficulties in the future.

## Methods

**Fig. 1:**
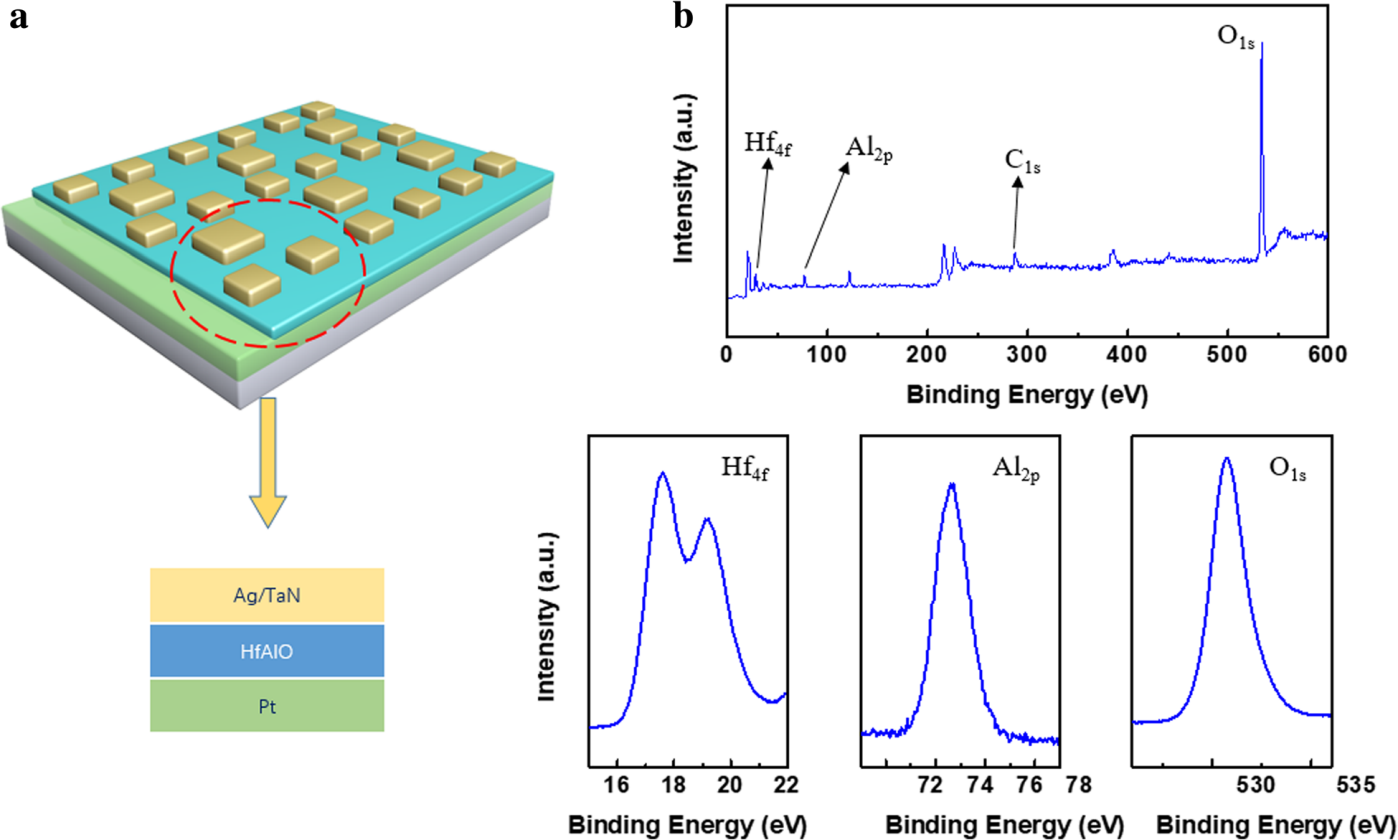
**a** The schematic of Ag/HfAlOx/Pt and TaN/HfAlOx/Pt devices. **b** XPS spectra of the 16-nm HfAlO

In this work, Ag/HfAlOx/Pt and TaN/HfAlOx/Pt devices were fabricated, respectively. The schematic is shown in Fig. 1a. First, a 70-nm-thin film Pt bottom electrode was deposited by physical vapor deposition (PVD) on the cleaned SiO_2_/Si substrate. Then, a binary high-k oxide HfAlOx film with a thickness of 16 nm was deposited using ALD derived from tetrakisethylmethylamino hafnium (TEMAH), trimethyl aluminum (TMA), and H_2_O precursors at 240 °C. Finally, 50 nm Ag or TaN top electrode film was fabricated by photolithography and fabricated by PVD. With bias on top electrode and ground on bottom electrode, direct-current measurements of the devices were performed by an Agilent B1500A semiconductor at room temperature. In addition, logic measurements were performed using an Agilent B1500A semiconductor device parameter analyzer and two semiconductor pulse generator units (SPGU).

## Result and Discussion

Memory and processor are separated in a traditional von Neumann computer architecture [17]. The transfer time of data stored in memory and calculated on the computing unit greatly limits the performance of the computer. It is possible to break the limitation by operating data directly on memory. The research of computing in-memory has the potential to break this limit.

**Fig. 2:**
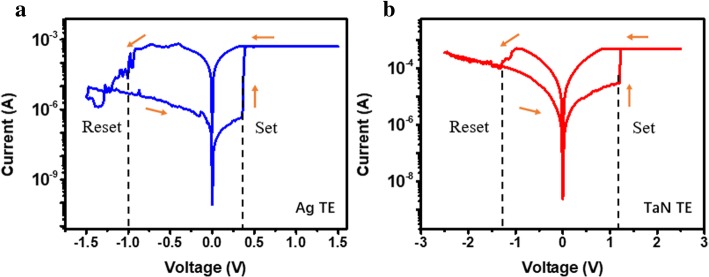
Typical current-voltage characteristics of Ag/HfAlOx/Pt (**a**) and TaN/HfAlOx/Pt devices (**b**)

**Fig. 3:**
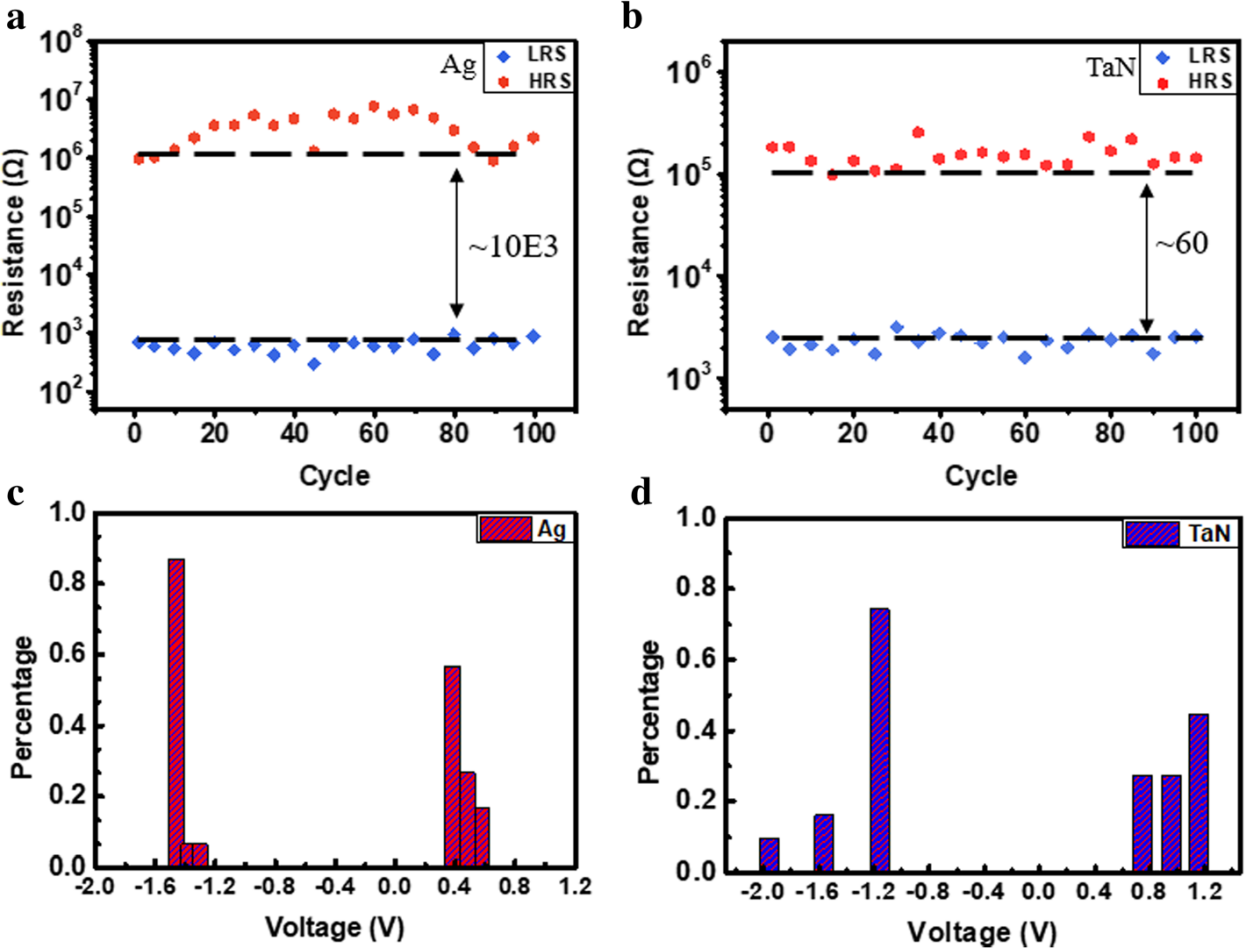
Endurance characteristics and set/reset distribution of Ag/HfAlOx/Pt (**a**, **c**) and TaN/HfAlOx/Pt device (**b**, **d**) under 100 consecutive DC sweeping cycles

To demonstrate the logic functions, RRAM was prepared with Ag/HfAlOx/Pt and TaN/HfAlOx/Pt. The schematic is shown in Fig. 1a; two small devices together with one large device form a minimum RRAM logic IMP logic unit. Different logic can be implemented by using multiple IMP cells. The 16-nm HfAlOx films grown by ALD were characterized by X­ray photoemission spectroscopy (XPS). As shown in Fig. 1b, the full XPS spectra and Hf4f, Al2p, C1s, and O1s are exhibited. From the XPS results, it can be concluded that the ALD HfAlO films have successfully been obtained. Figure 2a and b exhibit the *I*–*V* bipolar switching characteristics of the Ag/HfAlOx/Pt and TaN/HfAlOx/Pt measured by an Agilent B1500A semiconductor device parameter analyzer. The sweeping voltage was applied from − 1.5 to 1.5 V (for Ag) and − 3 to 3 V (for TaN) and a reading voltage of 0.1 V at room temperature. The resistance ratio of both Ag/HfAlOx/Pt and TaN/HfAlOx/Pt structures is shown in Fig. 3a and b. A device with Ag as an upper electrode can have a resistance ratio of 103 and TaN as the upper electrode can reach 60. Both Ag and TaN top electrodes exhibit superior bipolar switching characteristics. The distribution of set and reset operation voltage is presented as histograms in Fig. 3c and d, respectively. The Ag/HfAlOx/Pt devices exhibit much lower SET voltage. The performances of the two structures are compared. The SET and RESET voltage rang of the Ag/HfAlOx/Pt devices was from 0.33 to 0.62 V and from − 1.3 to − 1.5 V and the TaN/ HfAlOx/Pt devices was from 0.8 to 1.8 V and from − 1.3 to − 2 V. After comparison, it was found that the device using Ag as the upper electrode is more suitable as a device for implementing logic due to better stability and lower operating voltage.

**Fig. 4:**
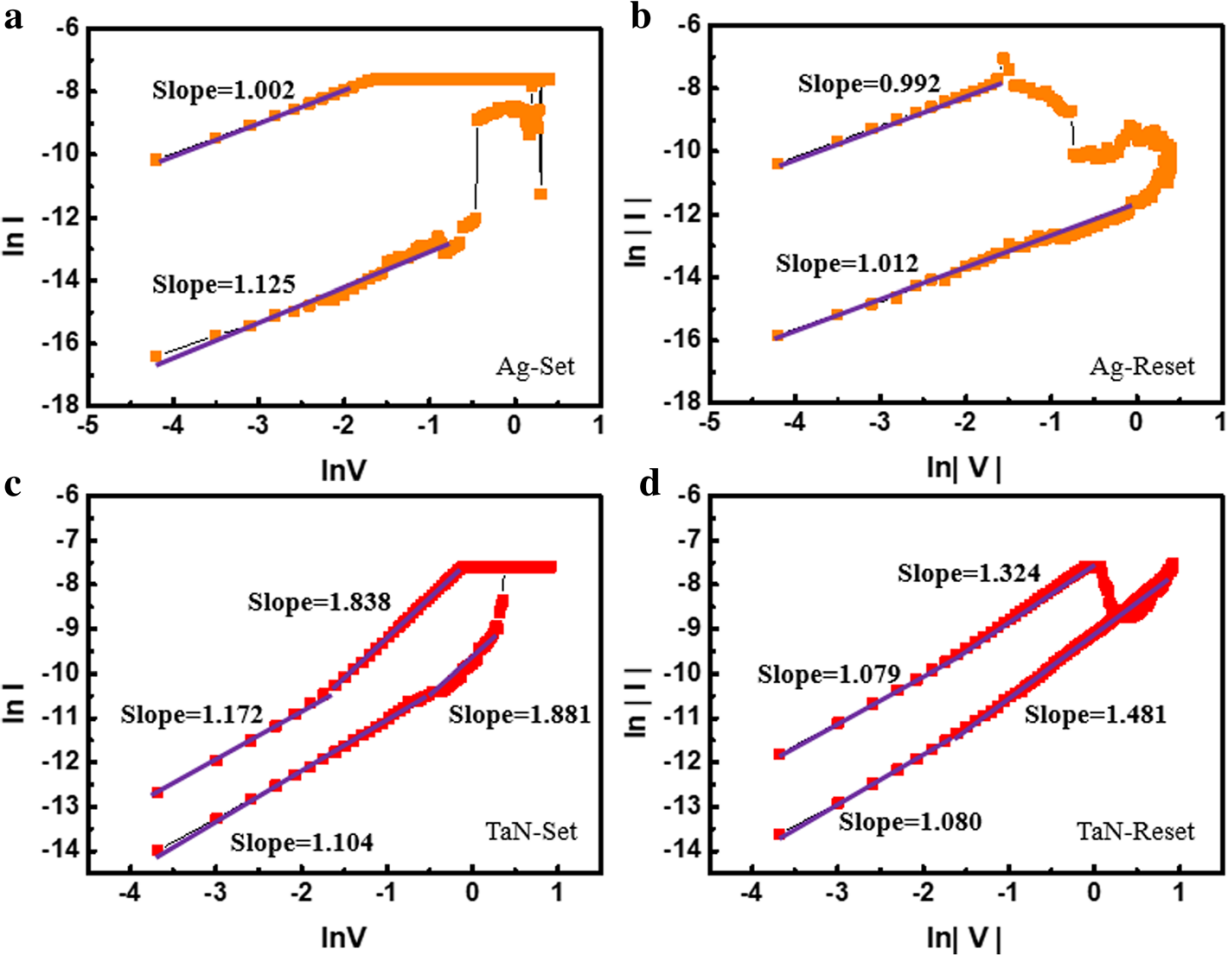
The current fitting of the Ag/HfAlOx/Pt devices under **a** positive and **b** negative electric fields and the current fitting of the TaN/HfAlOx/Pt devices under **c** positive and **d** negative electric fields

Moreover, the switching mechanism of the two types of structure is further expounded. The *I–V* curves are analyzed in Fig. 4a–d. The curves are taken in logarithmic coordinates to analyze the current status in the low-resistance state (LRS) and high-resistance state (HRS) states, respectively. It is shown in Fig. 4a and b the current transportation of Ag/HfAlOx/Pt devices exhibit ohmic current during the voltage sweeping. Whether applying a forward voltage or applying a negative voltage for TaN/HfAlOx/Pt devices shown in Fig. 4c and d, quasi-ohmic current (slope is approximately equal to 1) is presented in the LRS, while ohmic, quasi-ohmic, and space charge limited current is presented in HRS at positive electric field.

The reason for this phenomenon is that the resistance change mechanism of TaN/HfAlOx/Pt devices is due to avalanching generation and recombination of the oxygen ion and oxygen vacancy dielectric layer. In Ag/HfAlOx/Pt devices, the forming and rupture of conducting filaments, thanks to the redox reactions of metallic Ag, can be driven by a much lower electric field.

**Fig. 5:**
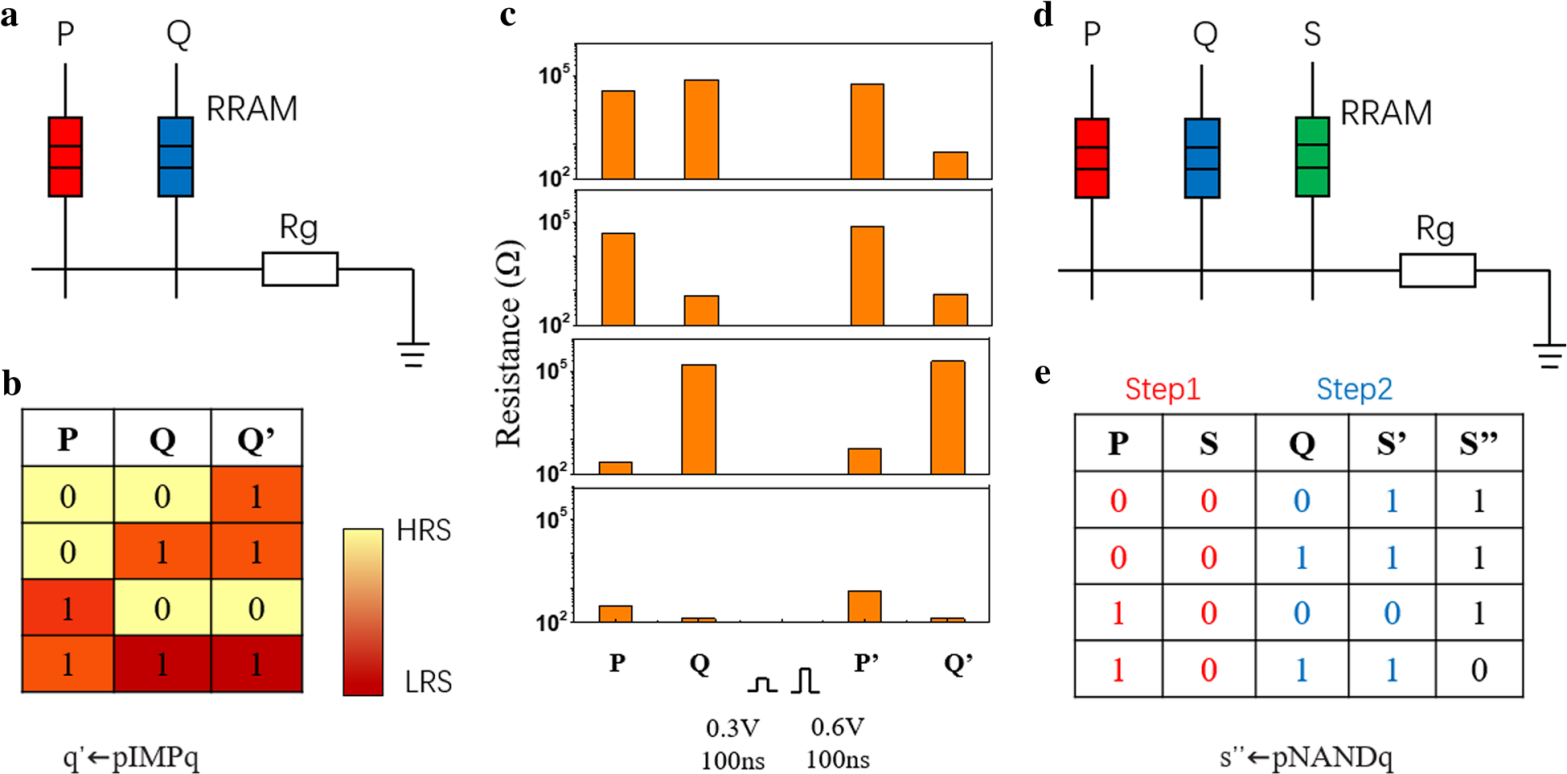
The test diagram of IMP (**a**) and NAND (**d**) logic. **b** The truth table for the operation q ← pIMPq (**c**) and q ← pNANDq (**e**). The state changes of P and Q with pulse (**c**)

In this experiment, low-resistance state (LRS) was defined as logic 1 and high-resistance state (HRS) as logic 0. The test diagram of IMP logic is shown in Fig. 5a. It is implemented by two RRAM devices P and Q and one fixed load resistor. The states of P and Q are represented by p and q, respectively. IMP is performed by two simultaneous voltage pulses: Va and Vb (we defined Va > Vset > Vb and Va – Vb < Vset so that Va could program logic 0 to 1 and Va − Vb could not program logic). The principle of logic p change is due to q. If q equals 1, then p is left unchanged because the voltage drop across p is nearly Va − Vb, and if q equals 0, the p is always equals 1. The truth table for the operation q ← pIMPq is shown in Fig. 5b and the state changes of P and Q with the pulse are shown in Fig. 5c. The NAND logic can be obtained through the two-step IMP logic. The implementation of NAND logic can be done by two-step IMP logic, because of the good uniformity. NAND is considered to be a universal logic, which means it can construct any Boolean logics through topologically connected NAND gates. As illustrated in Fig. 5d, the operation was implemented in a circuit with three RRAM devices: P, Q, and S. The inputs were the values p and q which were stored in devices P and Q. In the first step of the execution logic, S is initialized to a 0 state. Then, two steps of IMP were executed:

s′ ← pIMPs (1).

s′′ ← qIMPs′ (2).

The truth tables showing the equivalence of the sequence of operations to NAND are shown in Fig. 5e.

## Conclusion

In summary, two kinds of devices (Ag/HfAlOx/Pt and TaN/HfAlOx/Pt) were fabricated in this study. Both devices show superior switching characteristics. Ag/HfAlOx/Pt device has demonstrated advantages as a computing in-memory device such as CMOS compatibility, good uniformity, low operating voltage, and low power consumption. Logic was implemented through Ag/HfAlOx/Pt RRAM devices. The realization of low operation voltage computing in-memory devices provides an effective way to solve the traditional von Neumann structure difficulties in the future.

## Abbreviations

ALD: Atomic layer deposition
HRS: High-resistance state
LRS: Low-resistance state
